# “It is not by choice that I gave birth at home”: the social determinants of home births during COVID-19 in peri-urban and urban Kenya, a qualitative study

**DOI:** 10.1186/s12884-023-06038-x

**Published:** 2023-10-11

**Authors:** May Sudhinaraset, Rebecca Woofter, John Mboya, Sarah Wambui, Ginger Golub, Claire-Helene Mershon

**Affiliations:** 1 Department of Community Health Sciences, Jonathan and Karin Fielding School of Public Health, University of California, Los Angeles, Los Angeles, CA USA; 2 Innovations for Poverty Action, Sandalwood Lane, Nairobi, Kenya; 3 Jacaranda Health Solutions Limited, San Francisco, CA USA; 4 Bill & Melinda Gates Foundation, Seattle, Washington USA

**Keywords:** Home births, Birth, Kenya, COVID-19, Maternal health, Social determinants of health

## Abstract

**Background:**

The COVID-19 pandemic significantly impacted the provision of global maternal health services, with an increase in home births. However, there are little data on women’s decision-making and experiences leading up to home births during the pandemic. The objective of this study is to examine the economic, social, and health system factors associated with home births in Kenya.

**Methods:**

Community health volunteers (CHVs) and village leaders helped identify potential participants for an in-depth, one-on-one, qualitative telephone interview in Nairobi and Kiambu County in Kenya. In total, the study interviewed 28 mothers who had home births.

**Results:**

This study identified a number of economic, social, neighborhood, and health system factors that were associated with birthing at home during the COVID-19 pandemic. Only one woman had planned on birthing at home, while all other participants described various reasons they had to birth at home. Themes related to home births during the pandemic included: (1) unmet preferences related to location of birth; (2) burdens and fear of contracting COVID-19 leading to delayed or missed care; (3) lack of perceived community safety and fear of encounters with law enforcement; and (4) healthcare system changes and uncertainty that led to home births.

**Conclusion:**

Addressing and recognizing women’s social determinants of health is critical to ensuring that preferences on location of birth are met.

## Introduction

The COVID-19 pandemic has significantly impacted the provision of global maternal health services. Early estimates modeling the coverage of maternal and child services suggests a range of 8.3–38.6% increase in maternal deaths per month in low and middle-income countries (LMICs) [1]. Moreover, the burden of maternal mortality disproportionately occurs in developing contexts, with 94% occurring in LMICs [2]. In Kenya, studies found decreases in antenatal care use due to the COVID-19 pandemic [3], and estimates suggest an excess maternal mortality of 8.1% [4]. Additionally, there have been noticeable declines in institutional births in many parts of Africa [5–7] and subsequent fears that a shift towards home births will also result in increased maternal mortality [8, 9]. However, there are little data on how the COVID-19 pandemic has contributed to women’s decision-making and experiences with home births.

Studies have demonstrated the challenges in accessing broader maternal health services during the pandemic in Kenya [10]. The Kenyan Government responded quickly to the pandemic and enacted a number of preventive measures early, with the first case of COVID-19 detected on March 13, 2020, and implementation of strict preventative measures going into effect March 16, 2020 that included social distancing, school and higher learning institution closures, strict lockdowns, and nightly curfews that restricted movement within the country. While these measures aimed to reduce the spread of COVID-19, they also contributed to difficulties navigating to and from health centers. Existing studies demonstrate that structural level challenges, such as lack of access to transportation and fear of healthcare workers, contribute to women birthing in places where they had not intended to give birth [11]. During COVID, lack of emergency transport to health facilities due to government curfews and fear of contracting COVID-19 resulted in an increase in home births with the assistance of traditional birth attendants or midwives [10, 12].

Around the globe, data point to the various ways that COVID-19 has exacerbated existing class and gender inequities, with significant data suggesting that the pandemic has had disproportionate impacts on women and poorer groups [13, 14]. Broader social and structural factors have constrained women’s choices during the pandemic, with women oftentimes lacking decision-making power on where they give birth. Consequently, one useful approach to guide our understanding of how COVID-19 has influenced where women give birth is leveraging a social determinants of health perspective [15].

The objective of this study is to identify how broader social determinants of health influence women’s decision-making on where to give birth, including how these determinants may constrain women’s choices and decision-making power.

## Methods

### Social determinants of health framework

**Fig. 1:**
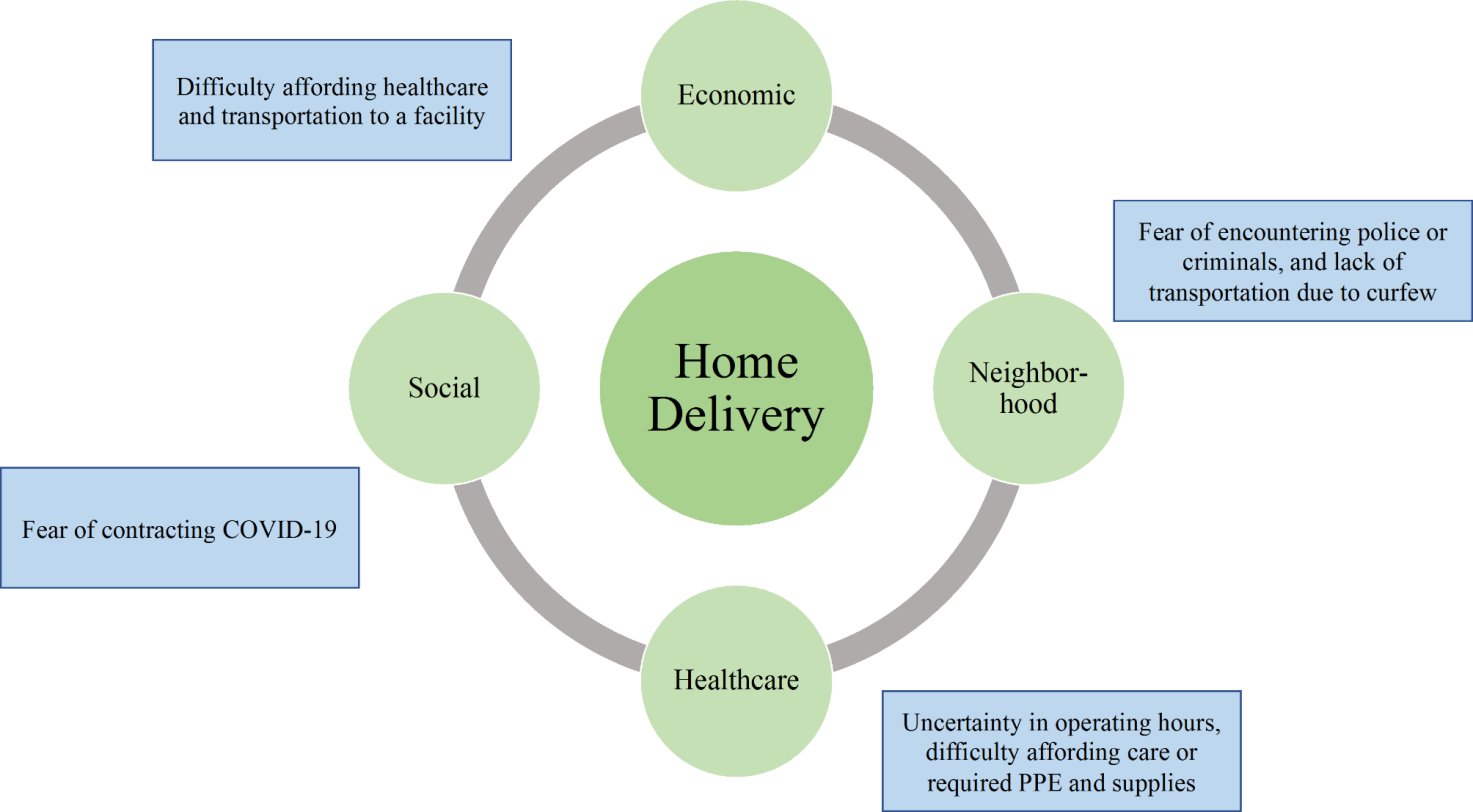
Social determinants of health for home births in Kenya during COVID-19

The social determinants of health framework provides a simplified model and identifies five major domains of health: (1) economic stability, (2) education access and quality, (3) healthcare access and quality, (4) neighborhood environment, and (5) social and community context. These five domains are the conditions where individuals live, work, and socialize that affect their healthcare and health outcomes [16]. Structural determinants generate or reinforce social stratification in a society and structure social groups within hierarchies of power, prestige, and access to resources (i.e. economic status) [16]. Within this framework, key axes of social stratification include income, education, occupation, social class, gender, and race/ethnicity. Structural determinants influence material circumstances (e.g., living and working conditions, food availability, neighborhood safety), behaviors and biological factors, and psychosocial factors, that ultimately impact health equity and well-being [16]. We adapt the SDOH framework to assess the economic, neighborhood, social, and healthcare system factors that influence women’s decision-making with and experiences of home births during the COVID-19 pandemic (Fig. 1).

### Study Design: study sample, recruitment and interview process

Women were recruited from the catchment areas of six health facilities in Nairobi and Kiambu Counties. Community health volunteers and village leaders were trained virtually on the recruitment scripts outlining the objectives of the study and the eligibility criteria for the respondents of interest. They were also trained on the safety measures against COVID-19 during the door-to-door recruitment. Through collaboration with community health volunteers (CHVs) and village leaders, contacts were collected among potentially eligible and interested women: those aged 15–49 years who gave birth since the first COVID-19 restrictions were put in place by the Government of Kenya (March 16, 2020), and who had a functional phone where they could be reached. The recruitment was done by the CHVs working in their respective community units/territories in the two study counties. Each CHV or village leader worked door-to-door in the units and collected contact information from eligible women. The community health volunteers then compiled the lists and submitted them to team leaders who collated and submitted them to the Institute for Poverty Action (IPA) research associate. This activity continued for one month. A separate study component administered quantitative surveys, after which a subset of women (n = 46, approximately 4% of the survey sample) who reported having a home birth during the quantitative telephone interviews were included in the qualitative sample. This study interviewed a total of 28 mothers for qualitative interviews.

One experienced, female qualitative interviewer was trained on the interview guide for 3 days, as well as on strategies for virtual interviewing such as building rapport by phone and effective probing techniques by phone (author and researcher SW). She piloted the tool on three women for three days to ensure the questions were well-understood by women, that the flow and length was appropriate, and to practice telephone interviewing. The interviewer called the potential respondents to confirm eligibility of home birth, obtain verbal consent which was recorded, and conduct a telephone in-depth interview lasting about 40 min to one hour to understand factors leading to her home birth and her overall birthing experience. The interviewer ensured that the participant had privacy and was alone before continuing on with the interview. The interviewer also ensured that the participant understood the study aims and the interviewers’ motivation for conducting the study at the start of each interview. The interviews were audio-recorded and the interviewer recorded field notes during and after the interview on any noteworthy themes or issue that may have arisen. Women who participated in the interview were provided Ksh 150 (approximately $1.40) in airtime to thank them for their participation. All participants were interviewed in Kiswahili.

### Analyses

All interviews were transcribed and translated into English. Four coders analyzed the transcripts using an emergent thematic coding approach through three phases of an iterative coding process. The individual who conducted all the interviews served as one of the coders and provided context and non-literal translation of quotes. Two coders were native Kenyans, and three lived in Kenya, while the fourth coder was in the United States. All coders had training in maternal health and qualitative research. Coding was conducted in Microsoft Word and Dedoose, a qualitative software. Questions, notes, and memos were kept in a shared Google Sheet document.

In the first phase of coding, two coders separately read and coded six transcripts, and a third coder reconciled the two drafts. This familiarized all coders with the transcripts and led to the first draft of the codebook. In the second phase, three coders split fifteen transcripts amongst themselves. The fourth coder reviewed all fifteen and made any necessary adjustments. Three transcripts were blind-coded by two coders during this phase to calculate inter-rater reliability, which was calculated as 80% agreement on those transcripts. The codebook was then updated again. In the third phase, one coder read and coded the remaining seven transcripts and finalized the codebook. With the final codebook, one coder re-read all transcripts to ensure the codes were properly applied. Between each phase, the entire coding team met to discuss questions and disagreements and decided on code application by consensus.

Throughout the coding process, transcripts were analyzed with particular attention to the circumstances that led to each woman giving birth at home. Coders created a codebook based on emergent themes from the interviews, rather than beginning with a priori ideas of factors that contributed to home births. Codes were categorized into sub-themes and then larger families. Examples of codes include “change in facility operations” and “couldn’t afford care” which were grouped into sub-theme “challenges due to COVID-19,” which was then categorized into broader family of codes that related to COVID-19. Once coding was finished, the team discussed themes across participants that resulted in each woman delivering at home. Themes were then grouped based on the Social Determinants of Health Framework. Data saturation was discussed and reached on these themes.

All study procedures were reviewed and approved by the University of California, Los Angeles (UCLA), Kenya Medical Research Institute (KEMRI), and research permits obtained from the National Commission for Science, Technology and Innovation (NACOSTI).

## Results

The results first describe the demographic of the study and unmet preferences regarding location of birth. The results then delve into the social determinants of home births including: (1) social factors– burdens and fears of contracting COVID-19; (2) neighborhood factors- lack of perceived community safety and fear of encounters with law enforcement; (3) economic factors- COVID-19 impacts on economic security; and (4) healthcare factors - access to maternity care, and healthcare system changes due to COVID-19 (Fig. 1).

## Demographic characteristics

Table 1 provides demographic information of participants. Of the 28 women interviewed, their ages ranged from 17 to 45 years, with an average age of 32. Only three had no previous births, while all others had 1–4 children before the most recent pregnancy. Sixteen women were married at the time of interview, while twelve were unmarried. The majority of participants had achieved less than some secondary school (57.1%) and were unemployed (57.1%). Among the 12 participants who were currently employed, nine worked in casual labor, one as a salary or contract worker, and two were self-employed. All but two women indicated that they experienced employment changes (e.g., loss of job or reduced wages) due to COVID-19. The mean score for food insecurity was 4.6 (range 0–6), indicating high levels of food insecurity in this population.

### Unmet preferences regarding location of birth

There were high unmet preferences regarding location of birth as all but one woman interviewed stated that she had planned to give birth at a facility (n = 27). Many women indicated that they specifically did not want a give birth at home or with a traditional birth attendant (TBA): “*It was very difficult…I delivered at the TBA and it was not my wish* (Age 45, 3 children)”. Even among those that gave birth with a TBA but did not prefer it, women shared how they came to do so. One woman stated: “*All my life I have believed in the hospital…that day I just had to deliver [with the TBA] because I had no other way*. (Age 34, 4 children)”. The one woman who planned to give birth at home did so because she was unable to afford the costs associated with a facility birth. When she arrived at the TBA’s house, the TBA encouraged her to go to the facility, but she could not afford to do so. She stated, “*You know the birth attendants sometimes get afraid of helping people give birth because of the complications that sometimes occur. So, he wanted to take me to the hospital. But within me, I just knew that my pocket does not allow me to go [to the hospital].* (Age 43, 5 children)”.

Notably, most women (n = 25) had a previous birthing experience, and all of them indicated previously giving birth at a facility. Only three women had ever given birth at home before. Several women described their aversion to giving birth at home. The majority had no prior home births, and only gave birth at home due to circumstances beyond their control. One woman succinctly described her feelings: “*It is not by choice that I gave birth at home. It is not something I would choose*. (Age 42, 4 children)”.

### Burdens and fear of contracting COVID-19 leading to delayed or missed care

The COVID-19 pandemic had dramatically impacted pregnant women’s lives even before they approached their delivery date. When the pandemic began in Kenya, women feared that they or a member of their family would contract COVID-19. This caused great strain for mothers and pregnant women, who worried both for themselves and their children. Of the women interviewed, nearly all reported fear of contracting COVID-19 (n = 24) and about one-third (n = 7) specifically reported fear of dying because of COVID-19.

One woman described the constant vigilance and concern for interacting with others: “*The challenge is that you never know the status of your neighbor, those coming in. In fact, you are never sure of the doctors….Right now, you are scared of all people. You cannot trust anyone*. (Age 30, 3 children)” Women also feared allowing others to interact with or hold their infants after birthing, which further increased social isolation and prevented social support women would normally have: *“Bringing up a newborn is difficult because you can’t give someone your baby to hold because they can contract COVID-19. You cannot go to social gatherings.* (Age 29, 3 children)” Half of the women (n = 14) reported such social struggle as a result of the pandemic.

Women faced social isolation due to their fear of interacting with others in public settings such as markets: “*You are scared even when you are moving around. You are not sure since you might contract [COVID-19] from the places that you go to. You might get it from the people that you greet. Maybe the person sitting next to you has it and you don’t know….* (Age 25, 4 children)”.

Nearly half of the women interviewed (n = 13) reported that they feared seeking healthcare because of the potential of contracting COVID-19. Although many facilities took precautions such as checking temperatures, requiring masks, and enforcing distance between patients, women maintained high levels of fear when seeking antenatal care: *“When it [COVID-19] struck, there was so much fear so when you walked to a place with many people you would feel that all of them are unwell…so sometimes you just had to stay at home and not leave.* (Age 45, 3 children)” Another women shared: *“Right now we are not free with the doctors…before when we went for clinic, we would queue on one bench and you are not afraid of the next person. You would ask questions about their baby and how she is doing, you could share a lot. Right now, people don’t want that. Everyone just wants to be on their own. You are not allowed to come very close and ask questions and challenges that your neighbor is going through…We don’t have freedom in our country or even at the hospital. (Age 30, 3 children)”*.

Ultimately, the fear of contracting COVID-19 also influenced women’s decisions to give birth at home. One woman stated, “*You know at that time, during the pandemic, people were afraid of others. At that point I decided to deliver at home because I might go to the hospital and contract the disease there and the child may get the disease too.* (Age 31, 4 children)” In this way, women had to navigate competing risks of giving birth at home or risking COVID-19 infection by leaving home and spending many hours at the facility. The social context of the women in this sample was impacted by the COVID-19 pandemic, reducing their social support and connection, and contributing to their home births.

### Lack of perceived community safety and fear of encounters with law enforcement

Community-level factors, such as lack of neighborhood safety, transportation, existing curfews, and perceptions of violent encounters with law enforcement, contributed to some women giving birth at home, particularly those who went into labor at night during the curfew hours. Nearly half of all women (n = 13) indicated that they were unable to access transportation due to the curfew. This meant that public means of transportation were nonexistent, taxis or motorbikes were not available, and that neighbors or friends who might normally be willing to drive them to the facility were afraid to do so. One woman described, “*Here if it reaches a time where people are not walking, even the motorist will not carry you because he knows he will be robbed…no one can offer to take you with their motorcycle, because you don’t know what you will meet ahead and what about when he comes back. If he comes back, he will be arrested by the police.* (Age 38, 5 children)” Similarly, “*Where I stay, there are no vehicles, no motorbikes, because the government directives also say that you should not be found outside from 10pm, so you can’t find any motorcycles.* (Age 29, 2 children)”.

Some women additionally feared leaving their homes at all because of a potential encounter with the police. For example, “*If there was a curfew, you should not be outside by 9pm, so there is no one you would call at night to walk with, and you could be found outside, beaten then arrested.* (Age 32, 4 children)” Even those who did not worry about a negative encounter worried about the time needed to provide a reason to police officers, and were thus dissuaded from leaving: “*If we go and get stopped by the police, by the time we explain it to them it may be a problem. I may deliver before I explain to them [why we are out after curfew].* (Age 45, 3 children)”.

Even women who lived within reasonable walking distance did not feel safe doing so due to perceived danger in the area, particularly because few other people were out on the street at the same time. For example, a number of women went into labor in the middle of the night and one described “*During the curfew hours you are afraid of walking alone because you may meet bad people.* (Age 45, 3 children)”. Some were afraid of encountering police: “*You know with curfew, people are afraid to go outside, either if you will meet a policeman who will beat you thoroughly, [or] you would meet a thief there*. (Age 38, 2 children)” This same woman goes on to describe: “*If curfew would not have been there, I would have gone. Now, curfew was there, and you don’t know who you will meet, you see people are in their houses…if it’s not the police who would beat you properly you would meet with a thief*. (Age 38, 2 children)” In these instances, the protocols that were developed to minimize the spread of the virus created perceived barriers to reaching healthcare facilities for labor and delivery. The neighborhood context of the women in this sample was such that they did not feel safe traveling through the neighborhood at night to get to the hospital, contributing to their home births.

### COVID-19 impacts on economic security and access to maternity care

Every woman interviewed (n = 28) reported job loss or reduction due to COVID-19 for either herself or her partner. Many were working in the informal sector either hawking or selling goods, or providing services such as cleaning and laundry. When the pandemic began, potential customers feared contracting COVID-19 and avoided markets or no longer felt comfortable allowing workers into their homes. Additionally, because schools were closed, some families had their children complete house chores and thus had no need for outside help. This led to all women in the sample having reduced or zero household income.

This reduction in wages led to high levels of food insecurity, with two-thirds (n = 19) reporting food insecurity: “*There is only a little to keep us going. Sometimes we eat, sometimes we don’t. We are not living well.* (Age 30, 3 children)” Women indicated that they struggled to eat enough while pregnant, and would sometimes forgo meals to allow their young children to eat: “*You are alone and all these three children should eat, you are pregnant and there is no job. If you do not rise up early and look for something, the children sleep hungry. Sometimes they sleep hungry and you wonder how your children are sleeping hungry and you are alive. It is painful.* (Age 32, 4 children)”.

Five participants reported difficulty affording healthcare during their pregnancies. When asked about seeking healthcare while pregnant, one woman stated “*I used to stay at home because I wasn’t feeling okay and I thought that I would be charged for treatment and I didn’t have money.* (Age 27, 4 children)” Despite free maternity services in public hospitals in Kenya, women described how they could not always afford the procedures or medications recommended by physicians: “*They just gave me prescriptions but I did not buy [them] because there was no money.* (Age 41, 4 children)” Additionally, facilities required women to provide their own supplies, which many could not afford due to the economic impacts of the pandemic.

When it came time to give birth, many women had no way to pay for transportation to a hospital for child birth such as a taxi (n = 5). One woman stated, “*I did not have any contact [for transportation] and there was no money. If it were during the day I would have walked. There was no money, and if you call a taxi you have to pay.* (Age 32, 4 children)” Other women may have been able to pay for a taxi, but were alone at the time and no transportation was available (n = 6). Often, a confluence of factors prevented women from accessing transportation: “*I couldn’t call my neighbors [to take me to the hospital] because where I stay, the neighbors wouldn’t have responded even if called…In addition to that, we had the curfew in effect and there were no taxis. Besides, I didn’t have any money.* (Age 24, 2 children)” In some cases, the TBA was closer than the hospital, which resulted in women seeking a TBA rather than attempting to reach the facility before giving birth. One participant noted: “*I had labor pains and there [were] no vehicles…So, I decided to go to the midwife [TBA]*. (Age 28, 3 children)” As a result, economic implications of COVID-19 directly led some women to give birth at home, either due to inability to get to the facility, or inability to pay for services at a facility. The reduced job opportunities and high level of food insecurity demonstrate the low level of economic security experienced by the women in the sample, which contributed to their home births as many could not afford to give birth in a hospital.

### Healthcare system changes and lack of certainty

Healthcare system factors led to home births in several instances due to a confluence of changes in protocols and uncertainty caused by the pandemic. Half of participants (n = 14) described lacking concrete information about when facilities were in operation due to healthcare worker strikes, shortages of supplies and staff, and curfew. In addition, due to protocols for distancing and not sharing spaces in the healthcare facilities, women experienced longer waiting times: *“In the past we used to go together and get served fast but now you can stay outside and they call one person…you can go at 8AM in the morning and come back at around 2 or 3 PM. In the past we used to get back at 9 or 11AM.* (Age 27, 3 children)*”* One woman succinctly described this issue: “*The hospitals were closed. Even if I went to the hospital, I wouldn’t be admitted because of [COVID-19]…so it was just me and my God.* (Age 24, 2 children)” Hearing stories from other women in the community who struggled to access healthcare facilities contributed to this belief for some women. For example, “*When you hear that COVID-19 has made [the hospital] closed and women are giving birth from outside at the gate, and I was due at that point, you know sometimes you lack directions. You don’t know what to do.* (Age 38, 2 children)” This ambiguity discouraged women from attempting to reach the healthcare facility for their births.

Indeed, because women were unsure when hospitals were open and accessible, many delayed or avoided seeking healthcare. Explaining this uncertainty, one woman remarked: “*Sometimes when you go to the hospital, you find that the doctors are few but the people are many at the hospital, or maybe the doctor has not come…COVID has made some to lose their jobs. You stay there for long and end up going back without being treated.* (Age 18, 1 child)” This also increased anxiety during pregnancy about if they would be able to give birth in the facility. “*I heard that the hospitals were closed, there was no getting in, so I was wondering how I will deliver and I became so stressed.* (Age 32, 4 children)”.

Women also reported some changes in protocols, such as reduced antenatal care and newborn check-up visits: “*I would [go to clinic] but not as much as with the first pregnancy. The first pregnancy I attended about six times. This one I went three times only because I started at three months, I was told to come back in February. When I returned in February, I was told to go back in April.* (Age 26, 2 children)” Facilities also limited the amount of recovery time women received in the facility after giving birth, down from the 24-hour minimum guidance: “*It was not the doctors’ wish to send people away but it was due to the regulations that no one was allowed to spend [the night] at the hospital, you just deliver and leave.* (Age 41, 4 children)” These changes in protocols may have added to the concern women had about whether they would be accepted to the facility, and may have decreased their opportunities to discuss birthing plans with healthcare providers during pregnancy.

Finally, one quarter (n = 7) of women interviewed did go to the facility prior to giving birth, but were turned away for different reasons. Two women described that although they were told they were not far enough along to be admitted, in fact they were quite close to giving birth: “*I felt like my time had come to deliver. It was around 2pm, 3pm. I went and sat at the gate and told them that I really feel like the baby is coming but they told me to go back home and come back at 11…I reasoned I may deliver outside there and the baby may die. So I took a motorbike and went back home. When I got there, it did not even take five minutes and I delivered.* (Age 34, 4 children)” Due to COVID-19 protocols that minimized the amount of time women could spend in healthcare facilities, they were unable to even enter the facility.

Two women were turned away from the facility because they did not have a face mask or other required supplies. One woman was initially turned away from the facility for this reason, and since it was late at night with curfews in place, there was nowhere to purchase a mask. Another woman described, “*I had nothing, I did not even [have] clothes to wear when going to the hospital to give birth. There at the gate we were told that we were supposed to have everything – the basins, clothes, diapers, and cotton wool – but I had nothing…When you would go, it was a must for you to enter with it in the gate and I didn’t have them. So, I was forced to persevere and give birth in the house.* (Age 27, 4 children)”.

Finally, two women were turned away because they could not afford care that the facility required before giving birth, such as an ultrasound scan or blood test. One woman stated, “*They wanted money, almost 4,000 [Kenyan shillings] before they could take the [blood] sample. I didn’t have any money at the time. That is when I decided to go back [home].* (Age 25, 4 children)” Given facility level changes that required fees for PPEs combined with increased economic insecurity due to the pandemic, women either did not seek healthcare or were unable to access healthcare. These healthcare factors impacted women’s access to healthcare facilities, discouraging women from attempting to go to the hospital to give birth, or resulting in some women being sent away from the facility.

## Discussion

This study identified a number of economic, social, neighborhood, and health system factors that were associated with a home birth. All but one woman indicated that she had originally planned to give birth in a health facility, but due to various reasons ended up birthing at home. Notably, even that one woman indicated economic reasons for planning a home birth, indicating that if she had more resources, she too would have preferred to give birth at a health facility. This study found that COVID-19 had wide-ranging effects that impacted women’s social, economic, and healthcare access. These critical social determinants of health, in turn, influenced women’s lack of ability to birth in a health facility, despite their preferences. While other studies have demonstrated the discordance between intentions and actual birthing location [17], this study provides further nuanced information on contextual circumstances that impeded women’s decision-making power and the wide-ranging consequences of the COVID-19 pandemic. Other studies highlight how social determinants of health may contribute to worse maternal and child health outcomes among vulnerable populations [14], highlighting the urgent need for resources to avert poor outcomes. These results point programs and policy makers to the types of resources and actions that may be needed for women in Kenya.

This study suggests that women often experienced circumstances beyond their control that led to choices that were contrary to their preferred mode of care. In many instances, women described the health facility as being inaccessible – whether that was due to lack of transportation, perceived violence that would occur if they left their house during curfew, actual hospital closures, or being turned away from the facility. These findings highlight that rather than lack of knowledge or intent to give birth at home, contextual circumstances driven by the pandemic were the driving factor in a home birth. Other studies conducted in Africa also found that fear of COVID-19 infections, economic challenges due to loss of jobs, and being turned away from the health facility limited maternal and neonatal health care access during the pandemic [12, 18].

Notably, the narratives in these interviews demonstrated how the social, economic, neighborhood, and healthcare factors that led to home births were intertwined. Economic factors, such as loss of employment for women or their partners, led to difficulty affording healthcare, or the required PPE related to COVID-19. This economic change meant that women and their families struggled to afford food and basic necessities. Further, when it came time to give birth, some women could not afford transportation. Accessing healthcare was also associated with new expenses, such as purchasing masks and other PPE. This has been shown to be associated with less likelihood to access antenatal care during the pandemic [3]. Finally, some women were unable to access healthcare since they could not afford required care or procedures. Some women could not afford birthing supplies required by some hospitals such as bleach, gloves, and PPE. This corroborates other studies which have found that the economic toll of COVID-19 on women, and in particular pregnant and post-partum women, has been catastrophic [19].

More broadly, COVID-19 influenced policies and protocols at the neighborhood- and healthcare facility-levels. The nightly curfew impacted women’s ability to access healthcare for their labor and delivery. Given that individuals were not permitted to be outside their homes at night, taxi and motorbike operators were often unavailable when they normally would have been present. Without this transportation, women were unable to access healthcare facilities. Further, enforcement of the curfew by police led to fear of violent encounters for women. Those who may have been able to access transportation even despite the curfew, or were within walking distance of a facility, were thus discouraged based on this fear.

Healthcare systems changed their protocols due to governmental and institutional policies, such as reducing the number of prenatal visits recommended or allowed, reducing the amount of time women could stay at the facility to give birth, and requiring PPE. Women thus spent less time with providers during pregnancy than usual, which may have impeded person-centered maternity care, including establishing patient-provider trust. Many women who did reach facilities were turned away due to new hospital policies, such as requiring women to bring their own masks and other supplies. Interestingly, another study in Kenya during the pandemic found more mixed results related to healthcare changes. Similar to our study, some women reported reduced number of prenatal visits and reduction in amount of time at the facility; however, the study also found that women reported improved perceived quality of care due to COVID-19 mitigation policies, facility cleanliness, and facility culture [18]. One reason that we may not have found these positive perceptions in quality may be due to our sample of women who gave birth at home; including women who also gave birth in hospitals may provide a more comprehensive understanding of health facilities during the pandemic. However, women in our sample did report on healthcare experiences during prenatal care visits.

There are strengths and limitations in the data worth noting. The women in this sample were at different stages of pregnancy when COVID-19 began, and those who gave birth in the first weeks of the pandemic may have different experiences from those who were newly pregnant. While we found consistency in our main themes, future research should consider these cohort differences. Second, this study includes a sample from peri-urban Kiambu and urban Nairobi county. These qualitative results are not generalizable to other parts of Kenya. Specifically, other studies have found that rural areas are more disadvantaged [20] as well as those in refugee and informal settlements [12]. Future studies should examine the social determinants of home births in rural settings and among vulnerable populations. The strength of this study is that it provides nuanced data among those who had home birth experiences at the height of the COVID-19 pandemic from a social determinants lens.

This study offers a number of policy and programmatic recommendations and areas for future research. First, this study points to the critical nature of addressing women’s social determinants of health and promoting gender intentional programming. Improving the daily living conditions, such as setting up food, economic, or unemployment aid, particularly in the perinatal period, is essential in promoting health equity. Additionally, addressing costs for maternal and newborn care, such as for commodities, PPE, and transportation are important factors in addressing access issues. Future research should focus on understanding the needs of women who generally have access to facility births, including those who gave birth in a facility for a previous birth, and choose or are forced to give birth at home subsequently. While birth plans are useful in facilitating women’s intentions and preferences, this study highlights the complex ways that women’s birth intentions may change. Training a healthcare workforce that understands the complexity of social determinants of health is important in meeting the needs of women and highlighting areas where intentions and preferences may not be met. This includes counselling women during antenatal care on birth planning, identifying appropriate support persons, identifying where to get supplies required to give birth at home or in the facility, and developing alternative strategies in instances where plans may change. Lastly, our study highlights the importance of community-based strategies and continuity of care. Future efforts should leverage robust community health strategies by engaging community health workers, including traditional birth attendants/midwives, to facilitate continuity of care, recognize early signs of complications, and ensure appropriate information is given to women and communities throughout pregnancy and postpartum to adjust plans when circumstance require it, or for awareness of social and economic supports that may be in place.

## Conclusions

Despite free maternity services in public hospitals in Kenya, this study found that broader social determinants of health influenced women’s location of birth. Addressing and recognizing women’s social determinants of health is critical to ensuring that preferences on location of birth are met.

**Table 1:** Demographic characteristics of women interviewed

Variable	Percent (Number) or Mean
*Age*	31.8
*Parity*	3.2
*Married*	57.1 (16)
*Education Level*	
No school, some primary, primary, or vocational school	57.1 (16)
Some secondary, secondary, or college	42.9 (10)
*Employment Status*	
Full-time	10.7 (3)
Part-time	32.1(9)
None	57.1 (16)
*Occupation Among Those Currently Employed (n = 12)*	
Casual labor	75.0 (9)
Salary/contract worker	8.3 (1)
Self-employed	16.6 (2)
*Changes in Employment due to COVID*	
Loss of hours or decreased pay	40.7 (11)
Loss of job or decreased job security	66.7 (18)
Disruptions due to childcare	29.6 (8)
Increased responsibilities	18.5 (5)
No change	7.4 (2)
Was not employed before/during COVID	11.1 (3)
*Food Insecurity (range 0–6)*	4.6

## Data Availability

The datasets used and/or analysed during the current study are available from the corresponding author on reasonable request.
